# Decreasing barriers to the utilization of cryopreserved sperm in male cancer survivors: an expert review and guide

**DOI:** 10.1093/oncolo/oyaf280

**Published:** 2025-10-25

**Authors:** Megan V Alexander, Hailie Ciomperlik, Anna Claire Reynolds, Allyson Nevins, Luwam Ghidei, Jordan Kassab, Kevin Campbell, John Sullivan, Michael D Jochum, Laura Detti, Terri L Woodard, Larry I Lipshultz, Laurie J McKenzie

**Affiliations:** Department of Obstetrics and Gynecology, Baylor College of Medicine, Houston, TX 77030, United States; Department of Obstetrics and Gynecology, Baylor College of Medicine, Houston, TX 77030, United States; Department of Obstetrics and Gynecology, Baylor College of Medicine, Houston, TX 77030, United States; Baylor College of Medicine, Houston, TX 77030, United States; Department of Obstetrics and Gynecology, Baylor College of Medicine, Houston, TX 77030, United States; Department of Urology, Baylor College of Medicine, Houston, TX 77030, United States; Department of Urology, Baylor College of Medicine, Houston, TX 77030, United States; Department of Urology, Baylor College of Medicine, Houston, TX 77030, United States; Department of Obstetrics and Gynecology, Baylor College of Medicine, Houston, TX 77030, United States; Department of Obstetrics and Gynecology, Texas Children’s Hospital, Houston, TX 77030, United States; Department of Obstetrics and Gynecology, Baylor College of Medicine, Houston, TX 77030, United States; Department of Obstetrics and Gynecology, Texas Children’s Hospital, Houston, TX 77030, United States; Department of Gynecologic Oncology and Reproductive Medicine, University of Texas MD Anderson Cancer Center, Houston, TX 77030, United States; Department of Obstetrics and Gynecology, Baylor College of Medicine, Houston, TX 77030, United States; Department of Obstetrics and Gynecology, Texas Children’s Hospital, Houston, TX 77030, United States; Department of Gynecologic Oncology and Reproductive Medicine, University of Texas MD Anderson Cancer Center, Houston, TX 77030, United States; Department of Urology, Baylor College of Medicine, Houston, TX 77030, United States; Department of Obstetrics and Gynecology, Baylor College of Medicine, Houston, TX 77030, United States; Department of Obstetrics and Gynecology, Texas Children’s Hospital, Houston, TX 77030, United States; Department of Gynecologic Oncology and Reproductive Medicine, University of Texas MD Anderson Cancer Center, Houston, TX 77030, United States

**Keywords:** oncofertility, fertility preservation, sperm cryopreservation, sperm banking, male infertility

## Abstract

**Background:**

Sperm cryopreservation offers male cancer patients a critical opportunity to preserve fertility prior to gonadotoxic therapy, yet utilization of banked sperm remains modest, typically under 10%. A structured understanding of barriers across the cancer survivorship continuum is needed to support patients in utilization of cryopreserved sperm in alignment with their reproductive goals.

**Materials and Methods:**

This expert review synthesizes current evidence and clinical experience to explore the multifaceted barriers to cryopreserved sperm use and propose specific solutions. Key intervention timepoints are examined, including the post-treatment fertility return visit, long-term cryopreservation while remote from family building, readiness for family building, and posthumous considerations. We subsequently outline an optimal Oncofertility Patient Care Pathway.

**Results:**

Multiple clinical, psychosocial, and financial obstacles limit the transition from sperm banking to use. Approximately one-third of survivors do not attend post-treatment fertility return visits, reducing opportunities for counseling and longitudinal reproductive planning. Psychological factors—including fear of cancer recurrence and delayed readiness for family building—contribute to prolonged storage. Many patients remain underinformed about the efficacy and processes of assisted reproductive techniques, while financial concerns are significant. These intersecting barriers hinder use of cryopreserved sperm. Opportunities exist to intervene at key timepoints, as outlined in the Oncofertillity Patient Care Pathway.

**Conclusion:**

A multidisciplinary and structured oncofertility pathway, such as detailed in this review, is needed to support cancer survivors in achieving their reproductive goals. Enhanced counseling, technology-enabled follow-up systems, targeted psychological support, and policies promoting broader insurance coverage for assisted reproduction represent key strategies to overcome existing barriers.

Implications for Practice While it is standard of care for patients to be offered fertility preservation prior to initiating gonadotoxic cancer therapy, subsequent utilization rates in males are found to be modest, at less than 10%. This expert review identifies modifiable barriers to cryopreserved sperm utilization for pregnancy intent. We propose actionable solutions and outline an “Oncofertility Patient Care Pathway” to guide providers caring for the male cancer survivorship population.

## Introduction

Fertility preservation is an important aspect of quality of life for individuals undergoing gonadotoxic cancer treatment. Approximately 10% of individuals diagnosed with cancer in the United States are under age 45, and 53% are males.^1^ ­Fortunately, interventions such as early detection and novel therapeutics have reduced cancer mortality and improved survival rates over the last several decades.^2^ As these advancements continue, there is increased focus on the complex issues of cancer survivorship, including the reproductive ramifications of cancer therapy. Radiation therapy and many chemotherapeutic agents exert direct gonadotoxic effects.^3^ Mechanisms include structural and cellular damage to sperm via DNA intercalation and induction of apoptosis resulting in potentially irreversible damage to spermatogonia.^4^^,^^5^

The American Society of Reproductive Medicine (ASRM) and the American Society for Clinical Oncology (ASCO) strongly recommend healthcare providers discuss fertility preservation options, such as sperm cryopreservation, prior to initiating cancer therapy.^6^^,^^7^ This method of fertility preservation is feasible, with 89% of men and 80% of adolescents able to bank sperm.^8^ It is also effective, with 50% of patients achieving parenthood with utilization of cryopreserved sperm.^9^^,^^10^ Historically, only 1 in 3 males with a new cancer diagnosis received a referral for fertility preservation counseling, although referrals are improving secondary to recent society recommendations and effectiveness, as well as formalized clinic policies and partnerships between oncologic and fertility centers.^11–13^ Of the males referred to a fertility clinic, 11%-87% elect to bank their sperm, with rates highest in oncologic institutions with formalized fertility preservation programs.^11^^,^^13^^,^^14^ In the setting of appropriate support, patient interest in fertility preservation is clear.

However, the modest (less than 10%) utilization rate of cryopreserved sperm deserves attention.^15^ Notably, the low utilization rate is often in the setting of prolonged storage duration, in which patients continue to pay for storage.^15–17^ It is incumbent on the medical community to identify and assist in decreasing barriers to utilization of cryopreserved sperm and unintentional prolonged sperm storage, where modifiable. We present our data regarding utilization and latency to use of cryopreserved sperm at our institution and review the available literature. We discuss the challenges and propose solutions to decrease barriers to cryopreserved sperm utilization for cancer survivors.

### Limited utilization of cryopreserved sperm: a literature review

At our own institution, we conducted a retrospective cohort study to assess cryopreserved sperm utilization patterns amongst male patients who underwent sperm cryopreservation for a cancer indication across a ten-year period (1/2010-10/2020). Of 725 men who cryopreserved sperm for a cancer indication, only 7.5% utilized their ­specimen for pregnancy efforts. 14.8% requested specimen discard, and the vast majority, 74.8%, continued long-term storage, Older age categories, particularly those 36 to 40 years old were more likely to utilize their specimen. The mean time elapsed between storage and utilization for pregnancy was 37 months, with older age predicting shorter interval between initial storage and use (*r* = −0.55, *P* = <.001). Nearly half (10/24, 42%) of nonsurvivors had dispositioned their specimen to a surviving partner to allow for posthumous procreation.

Studies amongst other institutions demonstrate concordant findings, and rates have remained stable over time. A 2016 systematic review of 30 studies encompassing 11 798 patients found that the aggregated rate of use of cryopreserved semen was 8%.^17^ In this review, 1%-17% of patients were found to be azospermic after cancer treatment.^17^ For those who utilized their cryopreserved sperm for pregnancy, 49% achieved parenthood. 11% of patients died during the study period.^17^ In a separate 2024 systematic review and meta-analysis of 69 studies encompassing 23 178 patients who cryopreserved at least one sample, a 9% utilization rate was found.^15^ Fertility outcomes were reported as rates per cycle of assisted reproduction, with a 34% pregnancy rate achieved via *in vitro* fertilization (IVF) with intracytoplasmic sperm injection (ICSI).^15^ Mean latency from banking to utilization is often several years. Whereas mean latency to use was 37 months in our patient population, other studies report a higher mean of 51-60 months.^9^^,^^16^^,^^18^ Voluntary sample discard rates vary from 16%-23%,^15^^,^^17^ again in line with our findings.

The long-term storage rate approximates 75% (defined by continued storage across a study period, *n* = 11 studies),^17^ with most patients assuming long term storage costs out of pocket. Sperm banking for oncologic indications is of longer duration compared to other indications (eg, intended fertility treatment, prior to vasectomy),^17^ underscoring the significant value of this service that provides hope for future family building.^19^

### The oncofertility patient care pathway

**Figure 1.**
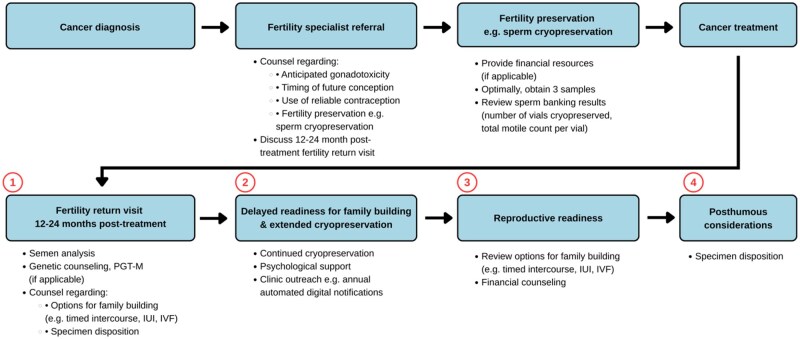
The oncofertility patient care pathway. Diagram illustrating an optimal oncofertility patient journey, including fertility preservation through subsequent follow-up care. Circled numbers correspond to timepoints at which patients commonly encounter barriers and represent opportunities for intervention. Abbreviations: IUI = intrauterine insemination; IVF = *in vitro* fertilization; PGT-M = preimplantation genetic testing for monogenic disorders.

The Oncofertility Patient Care Pathway (Figure 1) is initiated at the time of a cancer diagnosis. For men who have not completed family building or who are undecided, the potential reproductive impacts of the cancer diagnosis and associated treatment plan are discussed by the treating oncologist and/or at time of prompt referral to a fertility specialist (ie, reproductive endocrinologist or reproductive urologist). The option of sperm cryopreservation, including associated benefits, limitations, and patient concerns, is reviewed by the fertility specialist. If a patient elects to proceed with sperm cryopreservation, optimally three collections are processed prior to cancer treatment initiation.^20^ One to two years after cancer treatment, patients are scheduled for a follow up fertility consultation and post-treatment semen analysis. Depending on extent of sperm recovery and personal preferences, patients may elect to discard sperm or continue storage. Payment plans for the latter often range from one to ten years of storage time. Once ready for family building, options include spontaneous conception efforts or assisted reproductive means with intrauterine insemination (IUI) or IVF. In the event of patient death, surviving family may contact the cryopreservation bank to either discard or maintain samples in accordance with consent documentation at time of banking.

### Optimizing cryopreserved sperm utilization in cancer survivors

#### 1. Fertility return visit 12-24 months post-treatment

A follow-up appointment with a fertility specialist one to two years after completion of cancer treatment is recommended to reassess fertility potential, guide disposition of stored specimen, and provide fertility-related survivorship care counseling. This timing allows for possible interval sperm production recovery and also coincides with the American Urologic Association (AUA) and ASRM recommendation to delay conception for 12 months after gonadotoxic cancer treatment(s).^21^ Fresh semen analysis re-evaluates parameters such as volume, motility, and total count, which are influenced by baseline fertility, cancer type, and treatment regimen.^22^ Spermatogenesis recovery varies widely. For example, 64% of testicular cancer patients treated with platinum-based therapy demonstrate recovery at one year, and recovery rates in colon cancer patients treated with FOLFOX may exceed 90%.^23^ However, for men undergoing hematopoietic stem cell transplantation, recovery is less, estimated at 30%.^24^ Notably, there is a pressing need for human data on the gonadotoxicity of newer cancer therapeutics such as immunotherapy and targeted therapies.^25^^,^^26^

A post-treatment semen analysis enables counseling regarding risks and benefits of continued storage. Some patients decide to dispose of their specimen if testing reveals adequate sperm recovery.^27^ However, many will also elect to continue cryopreservation regardless of post-treatment parameters, citing fear of disease recurrence or to avoid “tempting fate.”^28^ In our practice, we support continued cryopreservation until patients have completed childbearing. Anticipatory guidance is also provided at this visit regarding options for future use of sperm for pregnancy attempts via IUI or IVF.

Unfortunately, approximately one-third of patients do not return for a post-treatment fertility consultation.^18^^,^^29^ This may be attributed to gaps in survivorship counseling, absent appointment reminders, and the fertility clinic’s lack of knowledge regarding oncologic treatment end dates. Patients who intend to continue cryopreservation (regardless of semen analysis results) are less likely to return.^29^ They may fear being pressured to discard samples^29^ or be unaware of broader counseling topics. Additionally, stigma and anxiety regarding fertility status, such as perceived threats to masculinity or fear of poor prognosis, contribute to nonattendance.^28^ Negative associations with the initial banking experience, often tied to the emotionally charged timeframe surrounding diagnosis, may also deter reengagement.^28^

A multi-pronged approach is recommended to support return. Fertility counseling should introduce the concept of the post-treatment fertility follow-up at the time of pre-treatment discussions. Oncology teams, including nurse navigators, can reinforce this recommendation during survivorship care and provide contact details for fertility clinics. An alert to the fertility clinic at treatment end enables the reproductive endocrinologist to facilitate appropriate follow up. Clinics can implement EMR-based reminder systems or leverage sperm bank billing notices as opportunities to prompt follow-up. Emphasis should be placed on the value of understanding fertility status and options for future family building with cryopreserved sperm, rather than solely on evaluating whether cryopreservation can be discontinued. Given prior findings, communication should avoid implying pressure to discard sperm in the case of recovered parameters.^28^ When feasible, offering virtual consultations or home-based semen collection may alleviate logistical and psychological barriers.^30^^,^^31^ While the period of cancer diagnosis and treatment planning will inevitably be an emotionally burdened time, sperm cryopreservation is an intervention that can instill hope for the future. We frame it as offering something to look forward to after a potentially arduous treatment journey.

#### 2. Delayed readiness for family building and extended cryopreservation

Age and life stage significantly influence the intent to utilize cryopreserved sperm. Among our own patient population, those older than 30 years at time of banking had utilization rates that exceeded 10% over a 10 year study period, compared to ≤6% among younger counterparts. Cancer survivorship itself also introduces physical and psychosocial burdens that may delay family building. Survivors often face residual fatigue and sexual dysfunction, which may impact their readiness to parent. Psychosocially, cancer-related anxiety, fear of recurrence, and altered self-identity can reduce or defer family building readiness. Financial toxicity resulting from medical bills, lost income, and delayed education exacerbates these delays, particularly in light of the high costs associated with assisted reproductive technologies (ART).^32^^,^^29^

Some patients express apprehension regarding childbearing secondary to potential health concerns in their offspring. Preimplantation genetic testing for monogenic disorders (PGT-M), when used in conjunction with IVF, may offer reassurance for those with hereditary cancer syndromes.^33^ However, we find that awareness of PGT-M remains low. We recommend that oncology clinics offer survivorship-stage referrals to genetic counselors, who can address inherited cancer concerns and guide patients toward appropriate reproductive care.^34^

Further interventions include patient education, psychological support, and systematic follow-up. Educational materials, such as interactive patient decision aids and ASRM/ASCO-approved videos, can address misconceptions about sperm viability, fertility timelines, and ART safety and efficacy (Table 1). Peer support groups and individual counseling may alleviate the mental health burdens that coexist with cancer survivorship and that impact readiness for parenthood (Table 1). To prevent loss to follow-up, contact redundancy on the part of fertility clinics is essential. Our institution’s banking consents require inclusion of secondary contacts, and a digital patient portal facilitates updates. These measures have allowed us to achieve a less than 5% lost to follow up rate (defined by inability to contact the patient to assess desire for continued banking after three documented attempts). Automated alerts can flag prolonged specimen inactivity and prompt personalized outreach or education about shipping options if patients relocate.

**Table 1.** Oncofertility patient resources.

	Resource	Link
**Peer support and counseling**	Cancer Support Community	https://www.cancersupportcommunity.org/
	CancerCare	https://cancercare.org/
	The American Cancer Society’s Cancer Survivors Network	https://csn.cancer.org/
**Fertility-related financial assistance**	Livestrong Fertility	https://livestrong.org/
	Expect Miracles Foundation	https://expectmiraclesfoundation.org/
	Worth the Wait	https://worththewaitcharity.com/
	Individual Cryobank-sponsored programs	See Cryobank website
**Educational materials**	Oncofertility Consortium	https://oncofertility.msu.edu/
	American Cancer Society	https://www.cancer.org/cancer/managing-cancer/side-­effects/fertility/preserving-fertility-in-men.html
	ASRM/SART Patient Education Short Videos	Via YouTube search

For those continuing long-term storage, specimen are often transferred to third-party facilities off-site from the fertility clinic. Patients can be advised that these companies, such as ReproTech and Fairfax, offer reduced prepaid pricing for long-term storage options. For example, annual rates approximate $400 for a 1-year plan, versus $1500 for 5 years or $3000 for 10 years of storage.^35^^,^^36^

#### 3. Reproductive readiness

Once a patient is ready to initiate family building, significant barriers may still impede utilization of cryopreserved sperm. While the costs associated with IUI (typically less than a thousand dollars for an unmedicated cycle) are relatively lower compared to IVF (ten to twenty thousand dollars or more per cycle), many patients elect for, or require, IVF to maximize chances of a successful pregnancy. IVF is recommended if sperm parameters are suboptimal (less than a 5 million total motile count),^37^ if a limited number of sperm vials are available, or if the intended father carries an inherited pathogenetic variant. Insurance coverage for assisted reproductive treatment remains inconsistent; fewer than half of US states currently mandate fertility coverage for iatrogenic infertility, and coverage gaps persist across self-insured employers and Medicaid-managed care plans.^38^ Patient awareness of existing coverage can be lacking. Clinics can encourage patients to verify their plan benefits and direct them to available financial assistance programs (Table 1). Policy advocacy at state and federal levels remains critical to expand fertility care access.

Misconceptions and psychological barriers may further delay use. Patients may incorrectly believe that frozen sperm is less effective than fresh, or express discomfort with the idea of assisted conception. Pregnancy outcomes of cryopreserved sperm are comparable to fresh sperm,^10^ and duration of storage does not appear to negatively impact success rates.^39^ When IVF/ICSI is used in conjunction with PGT, live birth rates may exceed those with spontaneous conception due to lower miscarriage risk.^40^^,^^41^

For patients who have relocated or lost track of their stored specimens, logistical and informational barriers may arise. Annual digital communications, such as summarizing specimen location, number of vials, and contact information, can mitigate this risk with minimal clinical burden. Cryobanks may also consider issuing digital “wallet cards” for storage information access. Patients can be reassured regarding the safety of specimen transportation should they relocate cryopreserved vials to alternate facilities.

#### 4. Posthumous considerations

Fertility preservation consents must include posthumous sample disposition. Options include discarding the sample or transferring ownership to a surviving partner to use for conception efforts. Nearly half of deceased patients in our institutional cohort requested posthumous transfer of sperm to a surviving partner. Yet only 60% of US clinics have policies addressing posthumous ART.^42^ It is imperative that clinics discuss and document sperm disposition intent at the time of banking, including provisions for death. Policies should clearly delineate the criteria for specimen release or continued storage, and legal consultation should be available as needed. Ethical considerations may arise in cases where family members, rather than partners, seek posthumous use, necessitating case-by-case evaluation.

## Conclusion

Sperm cryopreservation is an important consideration for cancer patients facing the potential loss of fertility.^3^^,^^4^^,^^5^ However, utilization rates of cryopreserved sperm remain modest. We propose an Oncofertility Patient Care Pathway to address challenges to cryopreserved sperm utilization and outline specific solutions. Our goal is to optimize patient counseling and improve reproductive decision making.

Health care systems must prioritize redundant communication strategies, involving both oncologists and fertility specialists, to ensure appropriate follow-up and education regarding options for utilizing cryopreserved sperm. Opportunities for reproductive counseling should be taken at multiple timepoints: at the time of cancer diagnosis, during survivorship care counseling, and at regular intervals post-treatment. A 12-24 month post-treatment fertility follow-up visit is of particular priority, due to the opportunity to provide extensive patient education regarding fertility status, PGT-M, assisted reproduction, and to address any possible misperceptions regarding these topics. Technology-driven reminders, such as automated alerts and EMR notifications from clinics and sperm banks, can further boost patient retention and minimize unclaimed sperm samples. Clinic policies regarding posthumous use of cryopreserved sperm and patient disposition is critical.

Psychosocial factors that impact cryopreserved sperm utilization deserve significant attention. Patient education and integration of mental health support, such as peer support groups and one on one counseling can address some of the stressors regarding future family building. Several states have passed legislation mandating insurance coverage of fertility preservation, and continued advocacy may alleviate some of the financial toxicity associated with fertility preservation. Current status of state and specific legislation for insurance coverage of fertility preservation can be found through the Alliance of Fertility Preservation (https://www.allianceforfertilitypreservation.org). Patient-directed education and financial assistance programs are outlined in Table 1.

Finally, the establishment of clear clinic policies regarding sperm disposition, including posthumous use, and the implementation of patient-centered educational tools will further reduce the uncertainty and stigma associated with sperm cryopreservation.

Institution-specific oncofertility care flowsheets, such as one based on the Oncofertility Patient Care Pathway depicted in Figure 1, can provide step-by-step guidance regarding timing of referrals and patient counseling. Alerting fertility clinics when cancer treatment concludes enables timely follow-up and post-treatment semen analysis. Post-treatment reproductive counseling facilitates informed decision-making regarding future fertility for cancer survivors. By reducing barriers along the Oncofertility Patient Care Pathway, we support cancer survivors in making empowered decisions regarding their reproductive future.

## Data Availability

The data underlying this article (specifically, our institutional data) cannot be shared publicly for the privacy of individuals that participated in the study. The data will be shared on reasonable request to the corresponding author.
